# Associational protection of urban ash trees treated with systemic insecticides against emerald ash borer

**DOI:** 10.3389/finsc.2023.990909

**Published:** 2023-02-09

**Authors:** Dorah M. Mwangola, Aubree M. Kees, Donald M. Grosman, Kari E. Norris, Mitchell P. Maddox, Brian H. Aukema

**Affiliations:** ^1^ Department of Entomology, University of Minnesota, St. Paul, MN, United States; ^2^ Arborjet Inc., Woburn, MA, United States; ^3^ Department of Chemistry, Bethel University, St. Paul, MN, United States

**Keywords:** emerald ash borer, agrilus planipennis, fraxinus spp., systemic insecticides, associational protection

## Abstract

Emerald ash borer (EAB), *Agrilus plannipenis* Fairmaire, is an invasive insect accidentally introduced to North America from Asia that attacks and kills ash trees (*Fraxinus* spp.). A common control strategy in urban centers has been the injection of systemic insecticides into mature trees, which can be costly at large scales. This study investigated whether treating a subset of a susceptible urban ash population could confer associational protection to untreated trees; i.e. improving or maintaining crown health of the latter. We selected approximately 100 mature ash trees along city streets in each of 12 sites in central and southeastern Minnesota in 2017. Each site had low but growing infestations of EAB such that canopy decline was not yet widespread. We treated 50% of trees with emamectin benzoate in eight sites and 50% of trees in four sites with azadirachtin in site-wide spatial gradients, such that the remaining 50% of trees at all sites were left untreated. Crown health of all trees was monitored for five years (2017 to 2021). Across all sites, we noted an overall maintenance or increase in crown health of both treated and untreated trees, while groups of untreated reference trees approximately three km distant from each site to monitor general tree health and EAB pressure declined quickly. These results suggested that protective benefits were conferred by treated trees to untreated trees within sites. Quantifying the spatial scale of canopy preservation of untreated trees within sites proved challenging due to the lack of variation in crown condition between treated and untreated trees. In two of the twelve sites treated with emamectin benzoate, we noted statistical evidence of improvements in crown condition of untreated trees when located within 100m of treated trees. Treating a subset of a susceptible ash population may aid in preserving untreated trees and provides a basis for developing a more cost-effective and environmentally favorable treatment regimen against EAB.

## Introduction

The increase in global trade and climate change has facilitated an increase in invasive species (1–4). Models investigating future global trends in invasive species predict an exponential growth in numbers (5, 6). Seebens et al. (2021), for example, estimated that the average number of invasive species would increase by 36% from 2005 to 2050 at a continental scale. Of the groups of invasive species analyzed, arthropods exhibited the highest increases. Invasive insects can cause ecological changes such as loss of genetic biodiversity at individual, population and community levels and can disrupt ecosystem processes (7–9). Moreover, damage caused by insects has adverse socioeconomic effects such as the loss of agriculture goods or recreational services provided by forests (10, 11).

Management of invasive insects is therefore crucial to limiting these adverse effects. Once the invasive insects have become established and are spreading, management options such as the use of chemical (application of insecticides), cultural (manipulation of forest composition and structure), biological (release of native insect enemies or microbial organisms) and mechanical (removal of trees, girdling of trees or tree parts) methods can be employed (12, 13). Although many of these management strategies can be effective, they are not without limitations. Management activities may exert non-target effects on native insects. Moreover, management activities impose additional economic burden to the costs incurred from damage. In the US, millions of dollars of annual federal and local government expenditures have been dedicated to invasive species management (13, 14). With continued increases in the numbers of invasive species and subsequent increases in ecological and socio-economic impacts of these species, there is a need to develop and employ management strategies that are sustainable and minimize adverse effects while being financially feasible (12, 15).

Emerald ash borer (*Agrilus plannipenis* Fairemaire, Coleoptera: Buprestidae) is an invasive insect that was accidentally introduced to North America from Asia on wood packing material in the 1990s (16, 17). The insect has since spread to 36 states in the USA as well as five Canadian provinces (18). In North America, emerald ash borer (EAB) attacks and kills healthy, native ash trees (*Fraxinus* spp.) and can attack and develop in fringetree (*Chionanthus virginicus* Linnaeus) (19–22). On ash trees, adult EABs emerge in midsummer and feed on ash foliage. Mated females oviposit in bark crevices and hatching larvae burrow through the bark to feed on cambial tissue, creating serpentine galleries that disrupt the vascular system. This feeding activity prevents movement of water and nutrients, which results in progressive crown thinning from the top of the tree downwards as larval densities increase before subsequent tree death within 1–5 years (23). Pre-pupae overwinter underneath the bark, pupate, and then emerge as adults (24–26).

Ash trees make up a substantial component of natural and urban forests in the USA. The progression of EAB over the past two decades has led to socio-economic impacts such as loss of a cultural and spiritual resource for Native American communities, financial losses to the wood industry, decreases in property values, and changes in plant and insect population compositions (27–30). Current management strategies for EAB include implementation of state quarantines, releases of biological control agents, and, in urban centers, tree removals or application of insecticide treatments (23, 31). Systemic insecticide treatments kill larvae feeding underneath the bark and adults feeding on foliage (32, 33). These urban management measures can exert exorbitant costs to governments and homeowners. The projected removal, replacement and treatment costs of street ash trees ranged from 0.3 to 0.9 billion Canadian dollars over a period of 30 years in 24 cities in eastern and western Canada, and an average of 10.7 billion US dollars over 10 years in 25 American states (29, 34). Additionally, adverse effects of insecticides on non-target organisms and the environment are a concern for some (35).

Finding a sustainable management option for EAB to alleviate costs while limiting environmental impacts is therefore crucial (36). Cost-benefit analyses of treatments versus removal and replacement have shown that treatments can be more economical (31, 37), and treating a proportion of infested ash can decrease density and dispersal (38, 39). A simulation analyzing management strategies to slow ash mortality in an urban setting showed that treating 20% of the trees annually ensured 99% survival of ash trees across 10 years (37). Proof-of-concept studies in state parks in Ohio and Maryland have showed protection of untreated trees in proximity to treated trees over three years (40, 41). Previous research regarding whether such strategies could be successful in urban environments where ash is a major component of the urban forest has focused on emamectin benzoate and imidacloprid; other insecticides such as azadiractin have not been tested in associational protection schemes (42). This study aims to test the hypothesis that treating a subset of an urban ash population (mature street trees) can confer protection to untreated trees. The specific objectives were to determine (i) whether increasing proportions of treated trees within different radii (10 m, 25 m, 50 m and 100 m) of untreated ash trees in urban centers could preserve or improve the crown health of untreated trees in areas of low but present emerald ash borer pressure, and (ii) whether emamectin benzoate *versus* azadirachtin is more effective in providing associational protection.

## Materials and methods

### Sites

**Figure 1:**
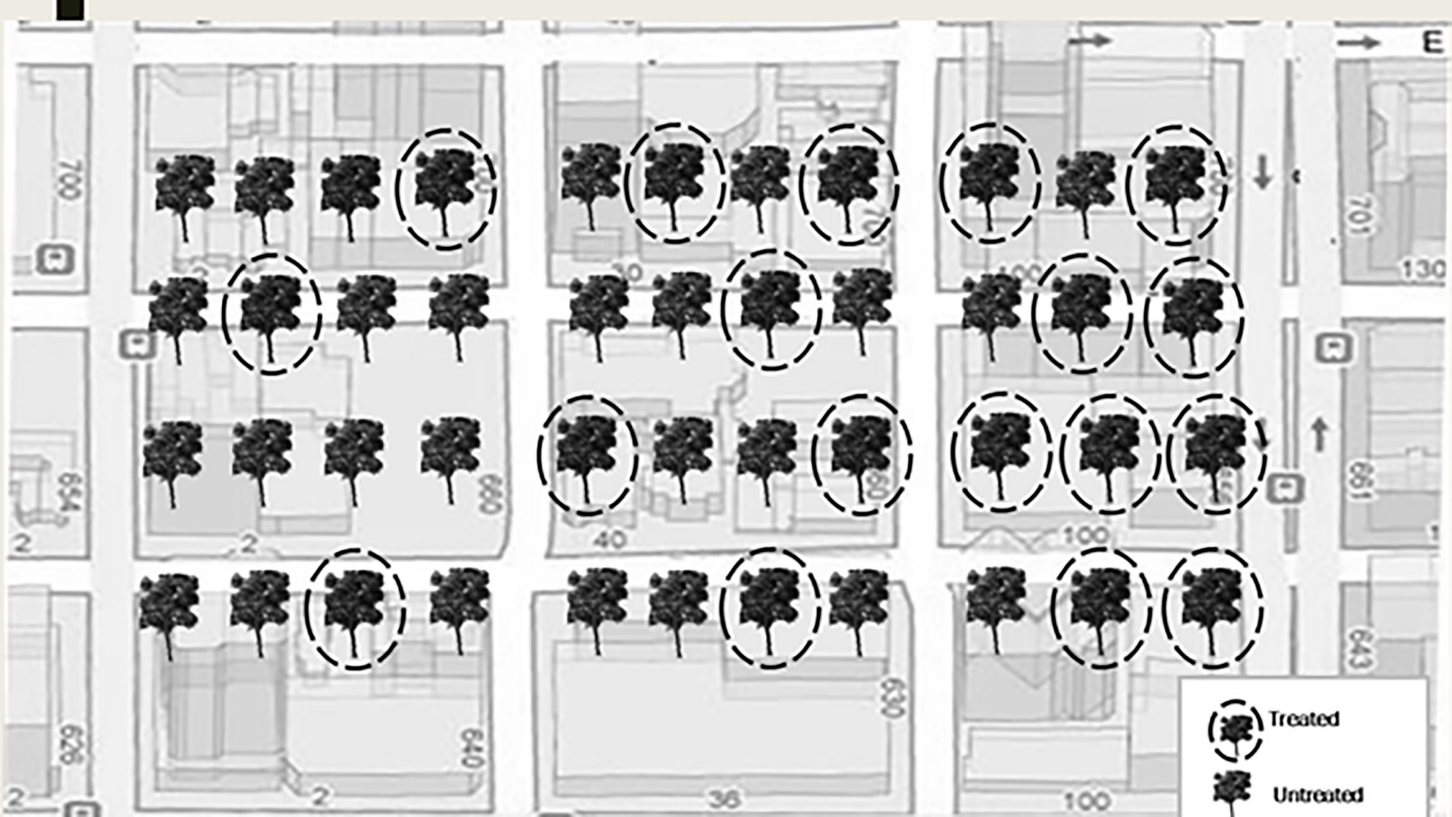
Schematic of treatment gradient of street ash trees. Each site had approximately 100 trees of which one half were treated. Trees and streets not to scale.

Twelve urban sites with trees with visible signs and symptoms of early infestation of EAB such as epicormic shoot growth and crown thinning were selected in Minnesota, USA in July, 2017 (**Table 1**). At each site, we selected approximately 100 mature green ash trees (*F. pennsylvanica*) with more than 70% crown present. Within each site, approximately 50 trees were injected with systemic insecticides at label rates of 0.2 g AI per 2.54 cm diameter at breast height (DBH) using a pressurized injection system (the QUIK-jet AIR^®^ tree injection system, Arborjet, Woburn, MA). Trees at eight of the twelve sites were assigned emamectin benzoate (TREE-äge^®^ G4, Arborjet, Woburn, MA; 395 trees injected total) while the remaining four sites, located a minimum of 300 m from four of the emamectin benzoate sites, received azadirachtin (AzaSol^®^, Arborjet, Woburn, MA; 200 trees injected total). A total of 678 trees were left as untreated controls across the experiment (i.e., half of the approximately 100 trees in each of the 12 sites). At each site, the trees assigned to the insecticide treatment were arranged in a spatial gradient from many untreated trees intermixed with a few treated trees to many treated trees surrounding only a few untreated trees (**Figure 1**). The mean (± SE) distance between trees at all sites was 9 ± 0.4 m. The mean DBH of all trees in the study sites was 44 ± 1.0 cm (i.e., approximately 14.9 m (2) of phloem per tree (43)).

**Table 1:** Experimental sites for study on associational protection from 2017 to 2021 at 12 sites distributed across 8 cities in Twin Cities metropolitan region and southeastern Minnesota, USA.

Site	City	Date of first EAB report	Experimental Sites			Reference Sites	
			Insecticide treatment	Mean (±SE) initial crown rating (2017)	Mean change in crown rating (±SE)	Mean initial crown rating of reference trees (2017)	Mean change in crown rating of reference trees
A	St. Paul	1/26/2009	Emamectin benzoate	9.1± 0.1	-0.2 ± 0.2	9.7± 0.2	-0.5± 0.3
B	Roseville	3/19/2013	Emamectin benzoate	8.3± 0.1	0.9 ± 0.2	8.8± 0.2	-5.8± 1.3
C	Rochester	8/8/2014	Emamectin benzoate	9.9± 0.0	0.0 ± 0.0	9.5± 0.4	-1.2± 0.3
D	Eagan	12/23/2014	Emamectin benzoate	9.7± 0.0	NA	9.3± 0.2	0.5± 0.1
E	Mendota Heights	5/12/2015	Emamectin benzoate	9.4± 0.1	0.3 ± 0.1	8.8± 0.3	-3.8± 0.3
F	Maple Grove	12/26/2016	Emamectin benzoate	9.5± 0.1	0.4 ± 0.1	9.6± 0.2	0.4± 0.1
G	Lake City	1/24/2017	Emamectin benzoate	9.7± 0.0	0.1 ± 0.1	9.3± 0.2	-4.3± 0.6
H	Coon Rapids	2/27/2017	Emamectin benzoate	9.4± 0.1	1.6 ± 0.1	9.0± 0.3	-0.7± 0.4
I	St. Paul	1/26/2009	Azadirachtin	9.4± 0.0	NA	9.7± 0.2	-0.5± 0.3
J	Eagan	12/23/2014	Azadirachtin	9.7± 0.0	0.1 ± 0.1	9.3± 0.2	0.5± 0.1
K	Maple Grove	12/26/2016	Azadirachtin	9.3± 0.1	0.6 ± 0.1	9.9± 0.1	0.4± 0.1
L	Lake City	1/24/2017	Azadirachtin	9.4± 0.0	-0.1 ± 0.2	9.3± 0.2	-4.3± 0.6

NA indicates sites removed from analyses due to pro-active tree removal by city staff before experiment conclusion or unexpected treatments of control.

Separate from the twelve experimental sites described above, a set of untreated, reference trees (*n*=10 trees at each city) were selected approximately 3 km from each study site. We refer to these trees as “reference” trees accordingly, with those in the previously described twelve experimental sites as “experimental” trees. The purpose of these “reference” trees and sites was to monitor EAB activity: similar to untreated trees within the experimental sites, we expected that these trees might succumb quickly in the absence of surrounding trees with tree injections.

Crown ratings of all trees at both experimental and reference sites were conducted every June (2017 to 2021) one week before any scheduled treatments (described below). Tree crowns were rated on a scale of 0 (dead) to 10 (100% canopy). Crowns were rated visually by three independent observers and then averaged. Precipitation, which could affect crown health was calculated as total monthly precipitation using daily precipitation and snow data at a representative site (Saint Paul, Minnesota) in 2017, 2018, 2019, 2020 and 2021 obtained from the National Oceanic Atmospheric Administration (NOAA) website.

Insecticide treatments in the experimental sites were implemented from 26 to 30 June 2017 and 31 July to 8 August 2017 for emamectin benzoate and azadirachtin, respectively. Treatments were reapplied according to the manufacturer’s guidelines with emamectin benzoate trees receiving treatments every other year, 26 to 28 June 2019, and azadirachtin every year, 4 to 8 June 2018, 10 to 17 June 2019 and 15 to 25 June 2020. Occasionally, we found that private citizens would arrange treatment of control trees in the public boulevard. In such instances, we switched the tree from “control” to “treated” in the dataset (*n*=28 trees at emamectin benzoate sites and *n*=20 trees at azadirachtin sites). Such trees were excluded in the pre-treatment analysis of median crown ratings at the start of experiment in 2017, but were included in analyses of associational protection at later stages. One site was removed from the experiment in 2021 when private citizens treated all control trees there in summer 2020. Another site, treated with azadirachtin, was lost when all but ten trees were removed by the city. After accounting for other tree removals across the remaining ten experimental sites, the total numbers of trees remaining in the study at the end of the experiment in 2021 were 158 azadiractin-treated trees, 342 emamectin benzoate-treated trees, and 435 untreated trees.

A linear mixed effects model (ANOVA) was used to analyze whether the initial crown conditions in 2017 varied across the two treatments as an initial quality control check. A second linear mixed effects model (ANCOVA) was used to examine how the changes in crown condition for 2017 (response variable) was affected by time (covariate) and treatment (factor). Terms for site and tree within site were incorporated as random effects. Finally, linear models were used to analyze associational protection for untreated trees in two ways. First, we examined associational protection within sites as follows. For each untreated tree, we constructed a distance-based neighborhood term that characterized the proportion of treated trees out of all trees within a given distance from that tree. We distance-based terms of 10, 25, 50, and 100 m radii separately for each site. We then used regression models to determine if the crown rating of untreated trees (response variable) changed with each of these terms (covariates) in separate models, i.e., did increasing proportions of treated trees affect the crown condition of a given untreated tree (**Table 1**). Second, we evaluated whether untreated trees declined at a slower rate depending on whether they were “reference” trees located approximately 3 km from the experimental sites vs. untreated “experimental” trees within the experimental sites. For this latter analysis, we fit an ANCOVA model using just untreated trees, and examined how the response, crown rating, varied with terms for time (covariate) and site type (reference vs. experimental, a factor).

Analytical assumptions of linear models such as homoscedasticity and normality of model residuals were examined through visual examination of residual plots. The best models were selected examining AIC scores in addition to examining significant of model covariates (*α*=0.05). All data analysis was conducted in R (package, *code*: lme4, *lme*) while making use of package tidyverse (44).

### Quantification of insecticide concentrations in ash foliage

Two to four weeks post treatment, leaves were harvested from branches approximately 6 meters high from five untreated trees and five trees treated with emamectin benzoate or azadirachtin, at each site, on 14 to 17 August 2017, 25 June to 5 July 2018, 8 to 25 August 2019 and 7 to 16 August 2020. The leaves were stored in Ziploc bags (26.8cm W x 27.3cm H) in a freezer at -20^0^C until insecticide concentration analysis using High Performance Liquid Chromatography – Tandem - Mass Spectrometry (HPLC-MS/MS). For analysis, five to seven grams of leaf material was weighed into beakers and then dried for 36 hours at 60°C. The leaves were crushed using glove covered fingers to sizes not larger than 2mm (2). Crushed samples were then transferred to the Q-Sep QuEChERS extraction kit (Restek Corporation, Bellefonte, PA), rehydrated with HPLC H2O water to a final mass of 10g and extracted using the unbuffered original protocol. Dispersive solid phase extraction (dSPE, Restek Corporation, Bellefonte, PA) was used as a secondary extraction before samples were loaded onto HPLC-MS/MS instrument.

Calibration standards for each active ingredient were prepared for the HPLC-MS/MS by diluting analytical grade stock solutions of each compound with untreated leaf extract, i.e., 6.2 µg/ml stock solution of emamectin benzoate (Sigma-Aldrich, St. Louis, MO) and 16.8 µg/ml stock solution of azadirachtin (Sigma-Aldrich, St. Louis, MO). A ThermoFisher UltiMate 3000 HPLC system and Bruker Amazon SL ion trap MS with an electrospray ionization source were used to detect the compounds. A 50 mm x 2.1 mm (3-μm particle size) Phenomenex LUNA Omega Polar C18 column with a guard column was used for separation. The methods used for optimizing separation of compounds in the emamectin benzoate and azadirachtin extracts are described by Zhou et al. (2016) and Pozo et al. (2003), respectively (55, 56).

The insecticide concentrations were determined based on their integrated peak areas and respective standard curves, and divided by the weight of dry leaves for each sample. The limit of quantification was 5 ng/ml for emamectin benzoate and 10 ng/ml for azadirachtin. Samples with concentrations above the limit of linearity (250 ng/ml) were diluted and reanalyzed.

## Results

### Tree crown condition and EAB pressure

**Figure 2:**
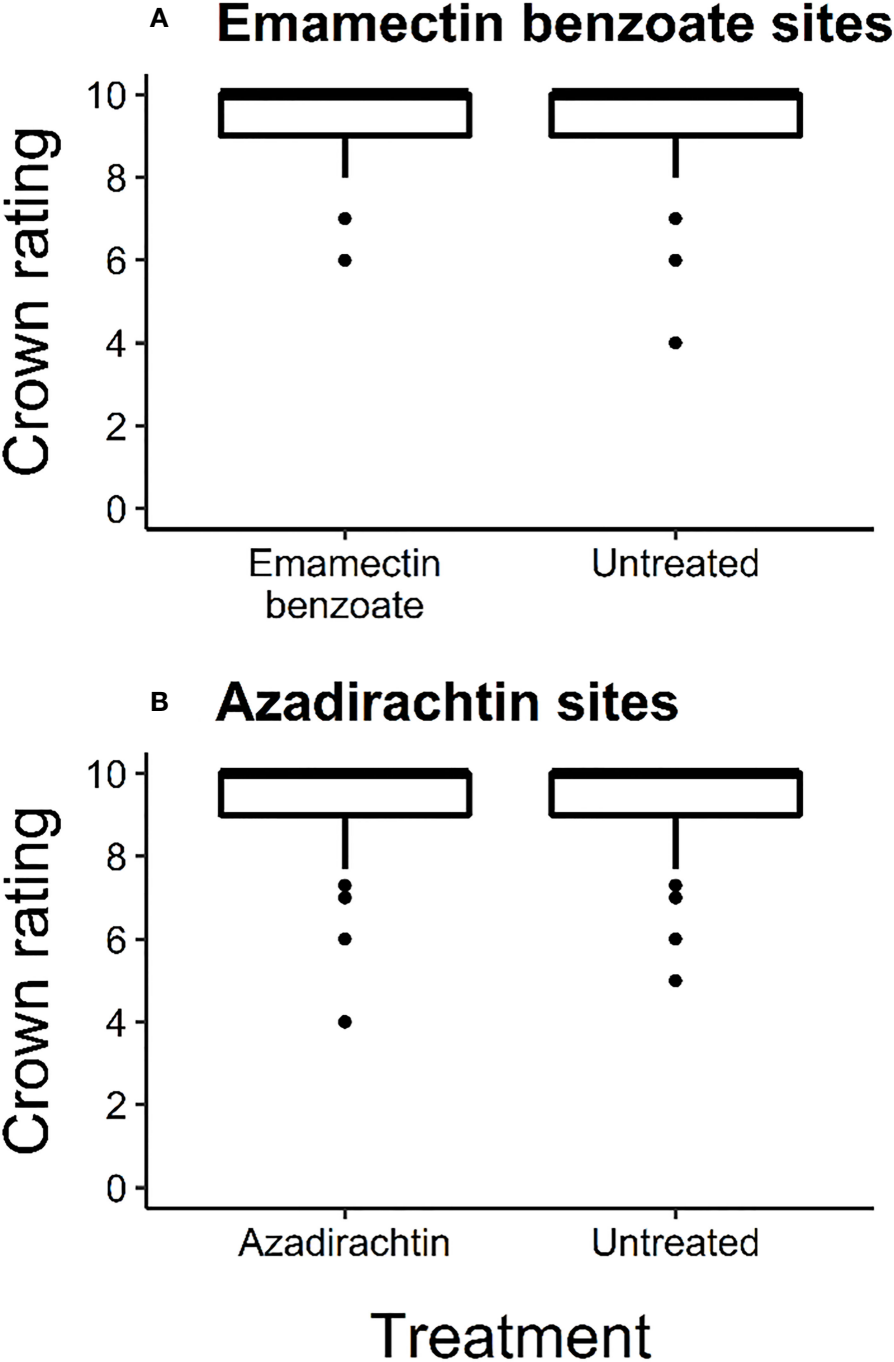
Box and whisker plot of initial crown ratings of ash trees in June 2017 before treatment with one of two insecticides, i.e., **(A)** emamectin benzoate (*F*_1,773_=0.11, P=0.74; n=8 sites with 383 treated and 392 untreated trees) and **(B)** azadirachtin (*F*_1,429_=2.23, P=0.14; n=4 sites with 189 treated and 242 untreated trees) in central and southeastern Minnesota, USA. Error bars show 95% confidence intervals about means. The upper whisker indicates the maximum value as the third quartile added to 1.5 times the interquartile range. The lower whisker represents the minimum value as the first quartile minus 1.5 times the interquartile range. The middle line of each box and whisker plot represents the median of the data set. Dots represent data points beyond plus or minus 1.5 times the interquartile range.

At the start of the experiment, most trees were in excellent condition as the median crown rating for all the trees across the experiment was 10 (**Figure 2**). The average ( ± SE) crown rating at that time for treated and untreated trees at emamectin benzoate sites was 9.39 (± 0.05) and 9.37 (± 0.05) respectively, and 9.46 (± 0.06) and 9.57 (± 0.05), respectively, at azadirachtin sites. Crown ratings at emamectin benzoate and azadirachtin sites were similar between treated and untreated trees (F_1,773_ = 0.11, *P=*0.74 and F_1,429_ = 2.23, *P*=0.14, respectively; **Figure 2**).

**Figure 3:**
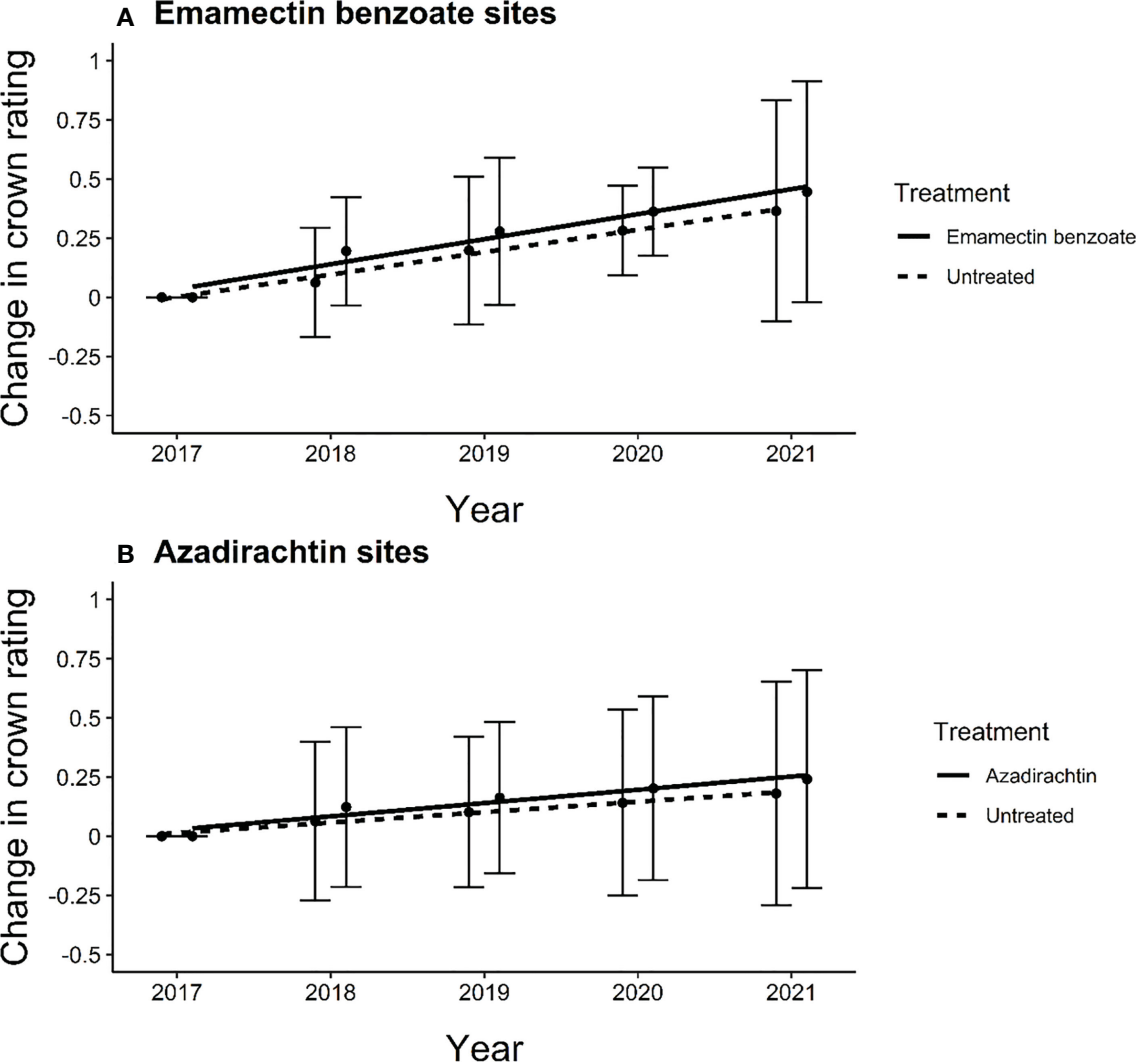
Regression line of average change in crown rating of ash trees from experiment initiation to 4 years post treatment in central and southeastern Minnesota, USA of **(A)** treated (y=0.0674 + 0.10766x) and untreated (y=0.0674 + 0.0498x) ash trees at emamectin benzoate sites and **(B)** treated (y=0.0550 + 0.10766x) and untreated (y=0.0550 + 0.04361x) ash trees at azadirachtin sites in 12 sites distributed across 8 cities. Error bars show 95% confidence intervals about means.

The crowns of the trees in our study sites proper did not decline over time. In fact, the average change in crown rating increased slightly from 2017 to 2021 for both treated and untreated trees at both emamectin benzoate and azadirachtin sites (**Figure 3**). In both types of sites, we noted gains between one quarter and one-half points, on average, across the five years of the study. The changes in mean crown rating over the five years of the study were similar between treated and untreated trees at both emamectin benzoate sites (F_1,8_ = 0.54, *P=*0.48) and azadirachtin sites (F_1,8_ = 0.87, *P*=0.38).

In 2021, at the conclusion of the study, up to eight untreated reference trees had been removed in each of five of the eight control plots set up 3 km away from each experimental site. At experiment initiation, in 2017, the average crown rating of the reference trees was between 8.8 and 10 in all eight cities. By 2021, the mean crown ratings remained at 10.0 ± 0.0 in one city and 8.8 ± 0.3 in another city. Mean crown ratings dropped to 8.3 ± 0.4 in two others, 7.5 ± 0.4 in one city, 5.0 ± 0.4 in two cities, and only 3.0 ± 2.0 in the final city. There, the trees outside our experimental site had degraded to very poor condition.

**Figure 4:**
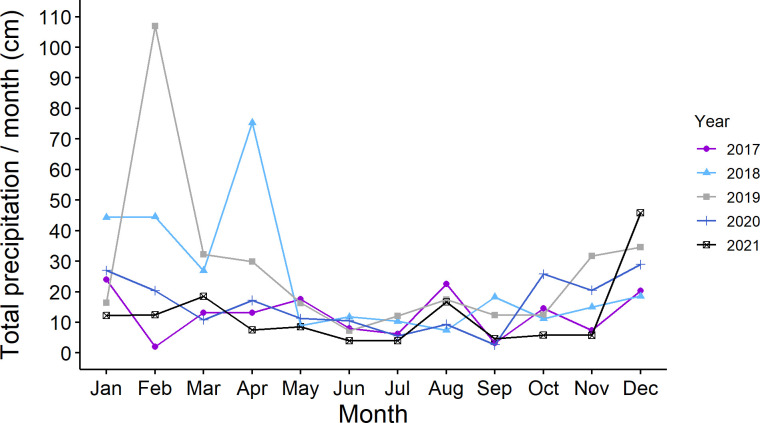
Total monthly precipitation (cm of precipitation/per month) in Saint Paul, Minnesota USA during five years of study (2017 to 2021).

We did not note drought conditions during the five years. A representative graph of total monthly precipitation in St. Paul, MN is shown in **Figure 4**.

### Evidence for associational protection

**Figure 5:**
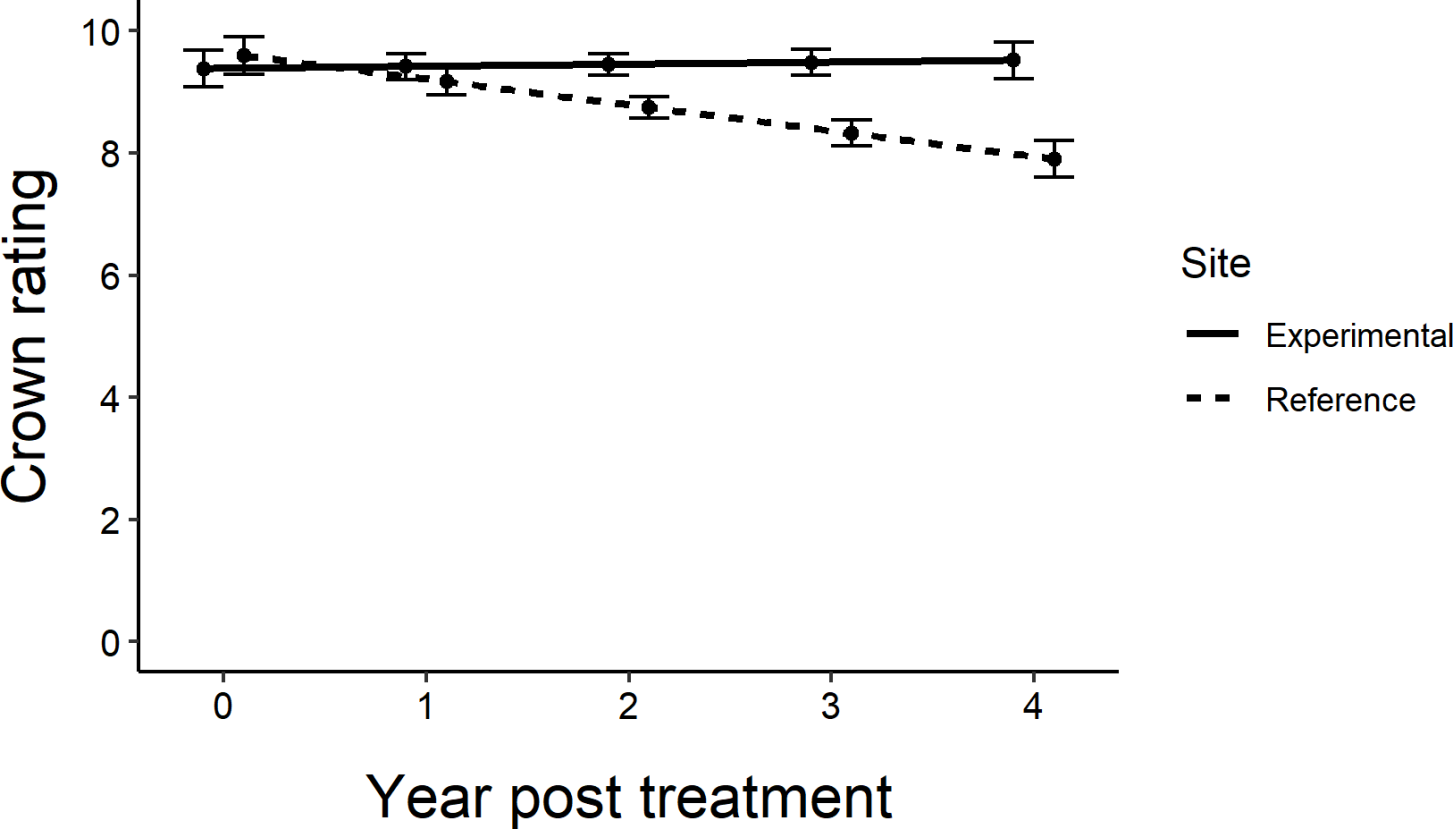
Regression lines of average crown ratings from experiment initiation to 4 years post treatment in central and southeastern Minnesota, USA for untreated ash trees in the experimental sites (where they were intermixed with a gradient of treated trees; Figure 1) (y=9.3825+0.0338x) vs. the reference sites located approximately 3 km away (y=9.5950-0.4238x). Only the line showing the decline in crown ratings for the reference trees has a slope that is statistically different from zero (F_1,76_ = 6.89, P=0.0105). Error bars show standard error of prediction.

Across all sites, the untreated reference trees declined more rapidly over the course of the experiment than untreated trees within the experimental sites (*F*_1,76_ = 6.89, *P*=0.0105; **Figure 5**). Within sites, as most of the trees remained in excellent condition, determining the distance over which associational protection might be acting or what proportion of trees should be treated to confer such protection to untreated trees proved challenging without strong variation in crown conditions (**Figure 3**). We observed statistically significant increases in crown ratings of untreated trees with increasing proportions of treated trees within a given radius from an untreated tree in only two out of twelve sites. That is, one could expect crown ratings to improve for an untreated tree if higher proportions of its neighbors within the nearest 25, 50, or even 100m in any study direction were treated (**Table 2**). The treated trees in both of these sites had received emamectin benzoate. The highest average increase in crown rating of untreated trees was observed in Coon Rapids (1.6 ± 0.1) and the lowest decrease was observed in the emamectin benzoate site in Saint Paul (-0.2 ± 0.2, **Table 1**).

**Table 2:** Model coefficients of regression equations for two sites in central Minnesota, USA where associational protection was detected at three different radii.

Site	Distance analysis	Intercept coefficient				Distance coefficient			
		Estimate	SE	t	*P*	Estimate	SE	t	*P*
Coon Rapids	25	1.13	0.29	3.89	0.0002	1.27	0.60	2.11	0.0030
	50	0.80	0.35	2.30	0.0239	1.88	0.69	2.73	0.0078
	100	-0.02	0.48	-0.04	0.9700	3.32	0.91	3.65	0.0005
Saint Paul	25	-1.11	0.24	-4.61	<0.0001	1.37	0.62	2.20	0.0303
	50	-1.37	0.29	-4.78	<0.0001	1.89	0.70	2.70	0.0081
	100	-1.50	0.36	-4.17	<0.0001	2.10	0.89	2.35	0.0206

For example, the top row, we found that improvements in canopy rating for an untreated tree could be modeled y=1.13 +1.27x, where x is the proportion of treated trees out of all ash trees that were treated with insecticide within 25 m.

### Insecticide concentrations in ash leaves

**Figure 6:**
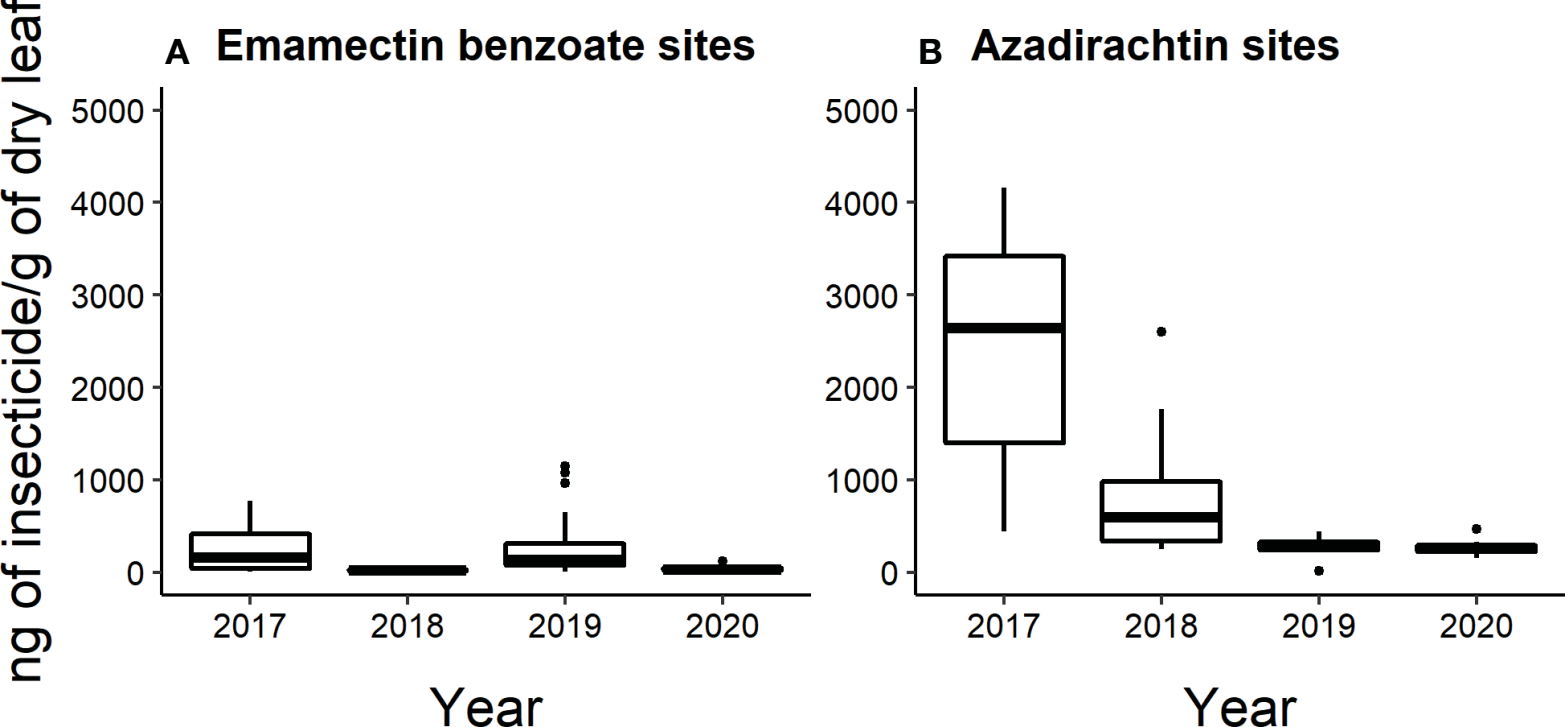
Box and whisker plot of the concentration of insecticide (ng of insecticide/g of dry flower) present in leaves collected from **(A)** emamectin benzoate-treated and **(B)** azadirachtin-treated trees determined by HPLC-MS/MS (n=5 trees per site per treatment per year). The data shown excludes values determined to be below the LOQ (Limit of quantification). Box and whiskers as described in **Figure 2**.

All leaf samples analyzed by HPLC-MS/MS collected from emamectin benzoate-treated trees in 2017 and 2020 (*n*=40 trees sampled per year) had detectable levels of insecticide. Whereas in 2018 and 2019, 26 out of 40 trees and 38 out of 40 trees had detectable levels of insecticide. Across the four years, the insecticide concentrations of the leaves collected from emamectin benzoate-treated trees ranged from 8.3 to 14,741 ng/g of dry leaf. Mean concentrations of emamectin benzoate were highest in the year that the trees were injected in the two-year cycles. That is, concentrations fell from 240 ± 35 ng/g of dry leaf to 25 ± 3 ng/g of dry leaf from 2017 to 2018 and from 636 ± 384 ng/g of dry leaf to 43 ± 3 ng/g of dry leaf from 2019 to 2020 (**Figure 6A**).

Approximately half of all the leaf samples collected from azadirachtin-treated trees each year had detectable levels of insecticide. In 2017, and 2018, 9 out of 20 trees had detectable levels; in 2019, 8 out of 20; and in 2020, 12 out of 20. Across the four years, azadirachtin concentrations in the leaves ranged from 12 to 4158 ng/g of dry leaf. The mean concentration of azadirachtin was highest in 2017 (2440 ± 404 ng/g of dry leaf), followed by 2018 (832 ± 225 ng/g of dry leaf)). In 2019, the mean concentration was 277 ± 31 ng/g of dry leaf and in 2020, the mean concentration was 278 ± 34 ng/g of dry leaf (**Figure 6B**).

## Discussion

Treatment of 50% of street ash trees preserved crown condition of untreated trees in our experimental sites as both untreated trees and those treated with emamectin benzoate or azadirachtin maintained or had improved crown ratings from 2017 to 2021 (**Figure 2**). To quantify the spatial scales at which such protective effects might be occurring within a site (i.e., 100m or less), there needed to be lower crown ratings in the less densely treated areas relative to the more densely treated areas within a site. The unexpectedly good condition of most trees from study inception (**Figure 2**) to study conclusion (**Table 1**, **Figure 3**) precluded the ability to quantify patterns further, as associational protection was quantifiable at within-site scales at only two sites (**Table 2**). There are two reasons, not mutually exclusive, why EAB densities at the experimental sites remained too low to cause crown decline overall (26).

The first reason is that the protective phenomenon worked over a broader spatial scale than the conditions we expected to test by arranging the injection schemes in a gradient spanning a few hundred meters across each site. Indeed, the maintenance of crown health in treated trees across all sites relative to observational reference trees located approximately 3 km from each site provides evidence to support this hypothesis (**Figure 5**). EAB was clearly present in the cities, as crown ratings decreased on the reference trees approximately 3 km from the site and many of these trees were removed due to infestation by EAB. It is possible that private treatment of random residential trees by homeowners inflated the numbers of treated ash in the area and contributed to a wider-scale associational protection, although visually the public mature ash trees outnumbered similar trees in residential backyards where other species were often planted. This study was conducted on public trees on boulevard rights of way, and we had no control over private landowner actions to treat trees on adjoining properties.

Second, cold temperatures during winter may have suppressed larval densities of EAB. In winter 2018-2019, for example, EAB larval mortality at Fort Snelling State Park, Sauk Centre, and Duluth, MN ranged from 40 to 99% mortality when air temperatures ranged from -29°C to -34°C (45). These observations are comparable with predicted cold mortality levels from laboratory and field studies of emerald ash borer conducted in Minnesota, i.e., 79% larval death at -29°C and 98% larval death at -34°C (46). Crown health ratings have been used as an effective measure of EAB population level, especially early in the invasion (47, 48). Canopy decline in green and white ash occurs when adult emergence levels range from 25 to 35 adults per m (2) of phloem and death of large trees (≥ 13 cm DBH) becomes noticeable when there are more than 100 adults per m (2) of phloem (26). We expect that climatic effects would have been uniform in both experimental and reference trees, however, given their proximity within approximately 3 km of each other in each city.

Although our results and others (40, 41) suggest that treating a subset of mature trees to provide associational protection to untreated trees is an ongoing, viable management strategy in early invasion stages with low densities of EAB, knowledge of the exact mechanism by which it works remains elusive. We hypothesize that the mechanism for associational protection is the reduction in EAB population from feeding on nearby treated trees and death of larvae in treated trees. Adult emerald ash borers will disperse about 100 m from an emergence point in an area with abundant ash trees (49) and protection of untreated trees within 100 m of treated trees (**Table 2**) suggests suppression at such distances. Emamectin benzoate is a neurotoxin that is lethal to emerald ash borer and kills both adults and larvae relative faster at lower doses than azadirachtin (50, 51). Azadirachtin disrupts larval growth and reduces fertility and fecundity of adult female EAB (32, 52). Our observations suggest that emamectin benzoate has a slightly more lethal impact on EAB populations than azadirachtin. We did not conduct analyses of phloem tissues where larval EAB feed, but leaf analysis suggested good but variable translocation of products into the canopies. The foliar concentrations of emamectin benzoate were highest in treatment years (2017 and 2019, **Figure 6**) and it is unclear whether extending treatments intervals to three years would yield similar associational protection. Temporal effectiveness declines over time without retreatment (41, 53).

Aside from studies on longevity, future work should refine the spatial, temporal, and EAB densities at which associational protection is effective. Previous studies and our own work have only examined distances ≤100 meters (40, 41). As we postulate that the associational effective range could be broader, further studies could confirm this possibility. O’Brien et al. (2017) showed that associational protection was effective in areas with low EAB densities (i.e., ≥ 75% ash survival at start of experiment) and ineffective in areas with high EAB densities (i.e., 25% ash survival at start of experiment). Branch and stem sampling could provide a more precise measure of EAB density (54). Determining the limit of effectiveness of associational protection should also incorporate additional insecticide treatment options. Although both emamectin benzoate and azadirachtin maintained or even improved overall crown health, temporal, spatial, and EAB density properties associated with azadirachtin schemes likely differ from those of emamectin benzoate. Incorporating all these aspects will provide case-specific treatment regimens using associational protection against this devastating invasive insect.

## Data availability statement

The raw data supporting the conclusions of this article will be made available by the authors, without undue reservation.

## Author contributions

DM and BA contributed to the conception and design of the study. DM, AK and DG performed field studies. MM and KN performed mass spectrometry analysis. DM wrote the first draft of the manuscript. All authors contributed to the article and approved the submitted version.
